# Differentiation of diffuse idiopathic skeletal hyperostosis (DISH) with back pain from back pain without DISH—clinical hints

**DOI:** 10.1016/j.ero.2025.03.004

**Published:** 2025-04-24

**Authors:** Amir Bieber, Reuven Mader, Shay Brikman, David Kiefer, Irina Novofastovski, Xenofon Baraliakos

**Affiliations:** 1 Rappaport Faculty of Medicine, Technion, Haifa, Israel; 2 Rheumatic Diseases Unit, Emek Medical Center, Afula, Israel; 3 Ruhr-University Bochum, Rheumazentrum Ruhrgebiet Herne, Germany

## Abstract

**Objectives:**

Diffuse idiopathic skeletal hyperostosis (DISH) is characterised by an exuberant new bone formation involving the entire skeleton and predominantly the spine. Clinical manifestations and symptoms that differentiate DISH from other chronic noninflammatory musculoskeletal diseases are still not well-described. This study aimed to compare the clinical presentation of patients diagnosed with back pain and the diagnosis of DISH vs those without DISH.

**Methods:**

Data on demographics, musculoskeletal symptoms and clinical examinations were retrieved from a database of 92 clinically symptomatic patients previously diagnosed with DISH and 41 patients with back pain, without concomitant DISH (non-DISH group). Differences between those measures were analysed.

**Results:**

Sex and age distributions were comparable between patients DISH and non-DISH. Chest expansion (CE) was smaller in DISH vs non-DISH (2.1 ± 1.2 cm vs 3.3 ± 1.3 cm, *P* < .001), whereas a cutoff of CE ≤2.5 cm was significantly associated with diagnosis of DISH (71.1% of DISH vs 31.7% of non-DISH patients, *P* < .001). In addition, occiput-to-wall distance (OW) was also significantly higher in DISH (5.2 ± 3.3) vs non-DISH (3.5 ± 3.7, *P* < .01). Compared with non-DISH, the odds ratio for being diagnosed with DISH were increased for patients with a combination of body mass index (BMI) <30, type 2 diabetes, CE ≤2.5 cm and OW ≥5 cm was 4.66.

**Conclusions:**

In comparison to non-DISH patients with back pain, the combination of high BMI, type 2 diabetes mellitus, small CE and high OW are highly suggestive for DISH patients symptomatic for back pain. Future studies of these measures are warranted in larger cohorts of patients and with DISH patients in various levels of evolvement.

**WHAT IS ALREADY KNOWN ON THIS TOPIC**

• DISH is characterized by exuberant new bone formation but manifestations for differentiation from non-inflammatory musculoskeletal diseases are unclear.

**WHAT THIS STUDY ADDS**

• In comparison to non-DISH patients with back pain, the combination of high BMI, type 2 diabetes, small chest expansion and high occiput-to-wall distance are highly suggestive for DISH patients.

**HOW THIS STUDY MIGHT AFFECT RESEARCH, PRACTICE OR POLICY**

• This study demonstrates that relatively simple clinical measures can significantly increase the odds ratio for identifying DISH.

Alt-text: Unlabelled box

## INTRODUCTION

Diffuse idiopathic skeletal hyperostosis (DISH) is a condition characterised by calcification and ossification of the entheses [1]. Classically, the disease affects the spine with a predilection to the thoracic spine, although other segments of the spine but peripheral joints and entheses may also be affected [2]. Although DISH, is a common condition, with prevalence ranging between 2% and 3% to more than 50% in specific populations, and has been described regularly for decades, the clinical manifestations are still not well defined [3]. It is, among other things, still debated whether DISH has distinct clinical features that enable its differentiation from other degenerative or even inflammatory conditions. It has even been suggested that patients with DISH have less spinal pain leading to clinical visits early in the development of their disease compared with patients with osteoarthritis (OA) of the spine, whereas other studies have been contradictory to this finding [4,5]. The correct description of DISH is important since, once diagnosed, appropriate measures to improve patients’ complaints can be initiated as early as possible in order to avoid complications (some of which might be life-threatening) such as spinal stenosis, sleep apnoea, complicated spinal fractures, difficult intubation or endoscopic procedures, swallowing difficulties and others [6,7]. The present criteria for diagnosing DISH are driven by imaging, whereas the findings are frequently accidentally observed.

In the present analysis, we explored the question on whether simple physical examination measures can increase the early suspicion for DISH in a daily practice setting compared with patients with back pain with (DISH) and without concomitant DISH (non-DISH).

## METHODS

This is a retrospective, cross-sectional, case-controlled study. The data set included patients with a previous diagnosis of DISH based on the Resnick and Niwayama criteria [8] and patients with back pain without concomitant DISH, which served as controls. DISH was determined using chest x-rays or/and thoracic spine computed tomography (CT); patients were enrolled in the Rheumatology Unit at Emek Medical Center, an academic hospital, between 2009 and 2016.

Data collection included patient's demographics comorbidities and constitutional measures, such as body mass index (BMI), waist circumference (WCIR), and the presence of type 2 diabetes mellitus (DM) and age at diagnosis of DISH. Localisation of all body areas of pain complaints was documented as well (shoulders, hands, hips, knees and legs). Spinal mobility measures included occiput-to-wall distance (OW) and chest expansion (CE) at the fourth intercostal space [9].

### Statistical analysis

Demographic and clinical characteristics of the 2 groups were compared by χ^2^ test for categorical variables and by *t* test or Mann-Whitney test in the case of non-normally distributed data for continuous variables. Multivariate stepwise logistic regression was performed with age at diagnosis. Furthermore, receiver operating characteristic (ROC) analyses were performed for the assessment of positive likelihood ratios for DISH.

## RESULTS

A total of 133 patients were included, 92 in the DISH group (29 [31.5%] men, mean age 64.1 ± 8.7 years, range: 40-84 years), and 41 in the non-DISH group (14 [34.1%] men, mean age 64.7 ± 8.7 years, range: 44-88 years). Demographic characteristics are shown in Table 1. There was a numerical difference between DISH and non-DISH regarding age at diagnosis, with DISH patients being older than non-DISH patients (60.5 ± 9.2 vs 57.8 ± 11.6, *P* = .17). In addition, patients with DISH had a significantly higher BMI (33.26 ± 5.77 vs 29.57 ± 4.67, for DISH vs non-DISH respectively, mean difference 3.69 kg/m^2^, SE = 0.86, *P* < .001) and WCIR (mean difference 8.5 cm, SE = 1.8, *P* < .001). DISH patients had a significantly higher frequency of DM compared with non-DISH patients (40.2% vs 14.6%, respectively, *P* = .004).

**Table 1:** Description of the 2 groups according to different variables

Patient characteristics	All (N = 133)	Study (N = 92)	Control (N = 41)	t/χ^2^	*P*	Effect size
Sex				1.27	.26	.098
Male	38 (28.6)	29 (31.5)	9 (22.0)			
Female	95 (71.4)	63 (68.5)	32 (78.0)			
Age	64.3 ± 9.1 (40-88)	64.1 ± 8.7 (40-84)	64.7 ± 10.0 (44-88)	−0.39	.70	.073
Age at diagnosis	59.6 ± 10.0 (23-86)	60.5 ± 9.2 (38-84)	57.8 ± 11.6 (23-86)	1.39	.17	.260
Age start complaint	48.7 ± 12.3 (15-82)	49.4 ± 13.0 (15-82)	47.0 ± 10.3 (18-72)	1.14	.26	.196
Years between start of complaint and diagnosis Median ± IQE	11.0 ± 12.3 (0-62) 6.5 ± 15.8	11.1 ± 11.8 (0-62) 7.0 ± 15.0	10.9 ± 13.5 (0-59) 6.0 ± 16.0	0.10	.92	.018
BMI	32.14 ± 5.70 (19-55)	33.26 ± 5.77 (22.6-55.0)	29.57 ± 4.67 (19-41)	3.57	<.001	.677
BMI>30 N, (%)	86 (65.2)	68 (73.9)	18 (45.0)	10.26	.001	.279
WCIR, cm, value ± SD (range)	104.6 ± 11.9 (73-148)	107.2 ± 11.7 (82-148)	98.7 ± 10.2 (73-122)	4.04	<.001	.758
Smokers N, (%)	42 (32.6)	30 (34.1)	12 (29.3)	0.29	.59	.05
Hyperlipidemia N, (%)	76 (57.1)	56 (60.9)	20 (48.8)	1.69	.19	.113
DM type 2 N, (%)	43 (32.3)	37 (40.2)	6 (14.6)	8.48	.004	.253
CE, cm, value ± SD (range)	2.5 ± 1.3 (0-6)	2.1 ± 1.2 (0-5)	3.3 ± 1.3 (0.5-6.0)	−5.34	<.001	1.00
CE≤2.5, N, (%)	79 (59.4)	66 (71.1)	13 (31.7)	18.85	<.001	.376
Occiput-to-wall distance cm, N, (%)	4.66 ± 3.48 (0-17)	5.17 ± 3.26 (0-16)	3.50 ± 3.72 (0-17)	2.61	.01	.491

BMI, body mass index; CE, chest expansion; DM, diabetes mellitus; IQE, internal quantum efficiency; WCIR, waist circumference.

For every year increase in age, the odds of being diagnosed with DISH increased by 5.9% (odds ratio [OR], 1.059; 95% CI, 1.013-1.107; *P* = .011). The age of complaint start was similar between the groups (49.4 vs 47, *P* = .26 for DISH vs non-DISH, respectively), and time from complaints to diagnosis was similar among DISH groups compared with non-DISH (11.1 vs 10.9 years. *P* = 0.1, for DISH compared with non-DISH, respectively). For every centimetre increase in WCIR, the odds of being diagnosed with DISH increased by 4.6% (OR, 1.046; 95% CI, 1.006-1.087; *P* = .023).

### Analysis of mobility measures between dish and non-dish patients

CE was significantly lower in the patients with DISH compared with patients with non-DISH (2.1 ± 1.2 cm vs 3.3 ± 1.3 cm, respectively, *P* < .001).

ROC analysis of CE revealed that the cutoff of CE ≤2.5 cm was indicative of DISH with high accuracy (73.9%, 95% CI: 66.4% to 80.5%), high sensitivity (71.7%, 95% CI: 61.4% to 80.6%), and specificity (76.8%, 95% CI: 65.1% to 86.1%) (Fig). OW was significantly higher with DISH (5.2 ± 3.3 cm) vs non-DISH (3.5 ± 3.7 cm, *P* < .01). Logistic regression using BMI (>30), type 2 DM, CE (≤2.5 cm), and OW (≥5 cm) revealed that each of DM and CE were independent significant predictors of diagnosing DISH (Table 2, Table 3).

**Figure:**
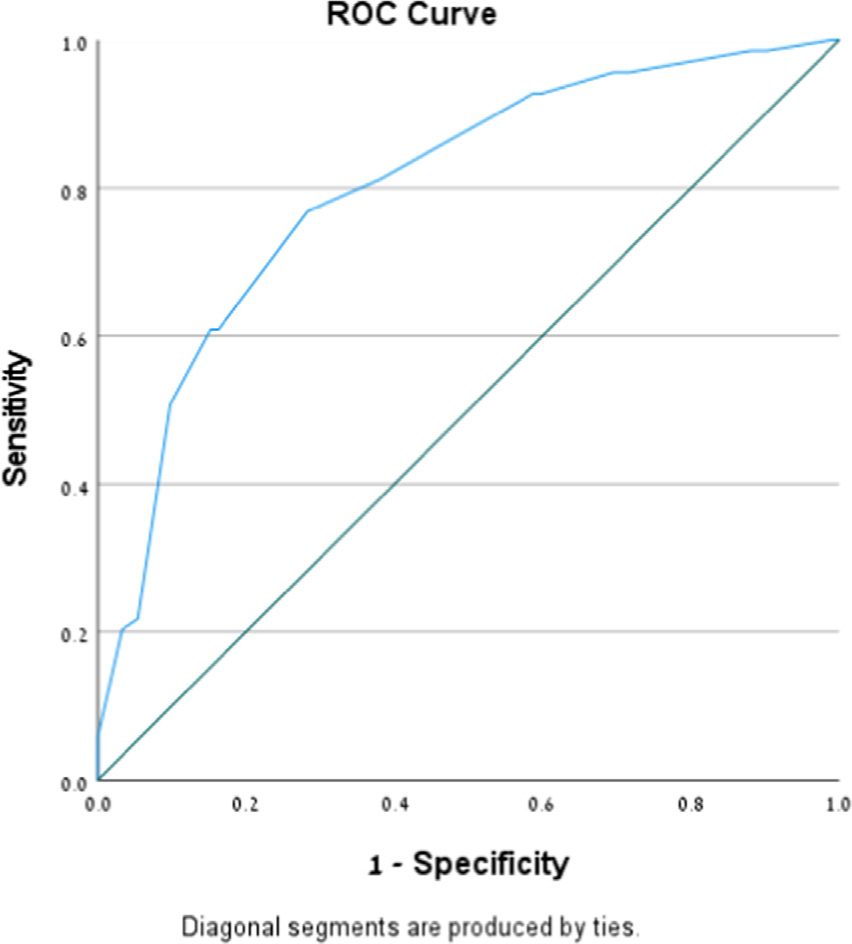
Receiver Operating Characteristic (ROC) curve for chest expansion (CE) and the diagnosis of DISH. Area under the curver = 0.801, with St error of 0.035. (bounds—0.732 to 0.87). DISH, diffuse idiopathic skeletal hyperostosis.

**Table:** 

**Area under the curve**				
Test result variable(s): CE				
			Asymptotic 95% confidence interval	
Area	Std. error^a^	Asymptotic sig.^b^	Lower bound	Upper bound

.801	.035	.000	.732	.870

The test result variable(s): CE has at least one tie between the positive actual state group and the negative actual state group. Statistics may be biased.				
a. Under the nonparametric assumption				
b. Null hypothesis: true area = 0.5

**Table 2:** Odds ratio of BMI, CE, OW, and DM for the presence of DISH compared with controls

Patient variables	Study	Control	OR	95% CI	*P*
BMI>30	68	18	3.46	1.59-7.54	.002
CE <2.5 cm	66	13	5.46	2.46-12.16	<.001
OW ≥5 cm	49	14	2.20	1.02-4.72	.044
DM	37	6	3.92	1.50-10.26	.005

BMI, body mass index; CE, chest expansion; DISH, diffuse idiopathic skeletal hyperostosis; DM, diabetes mellitus; OR, odds ratio; OW, occiput-to-wall distance.

**Table 3:** Logistic regression using BMI (≥30), type 2 DM, CE (≤2.5 cm) and OW (≥5 cm) for the presence of DISH

							95% CI for EXP(B)	
Patient variables	B	SE	Wald	df	Sig.	Exp(B)	Lower	Upper
DM	0.969	0.526	3.399	1	0.065	2.635	0.941	7.384
BMI	0.791	0.441	3.211	1	0.073	2.205	0.929	5.235
CE	1.169	0.468	6.238	1	0.013	3.218	1.286	8.050
OW	0.189	0.455	0.173	1	0.678	1.208	0.496	2.944
Constant	−0.589	0.374	2.476	1	0.116	0.555		

BMI, body mass index; CE, chest expansion; DISH, diffuse idiopathic skeletal hyperostosis; DM, diabetes mellitus; OW, occiput-to-wall distance.

The OR for having DISH among patients with BMI >30 and CE of less than 2.5 cm was 1.15-3.83, for patients with BMI ≤30, OW ≤2.5 cm and CE >2.5 cm was 0.87, and for all of those findings in combination: BMI ≥30, type 2 DM, CE ≤2.5 cm and OW ≥5 cm was 4.66 (with 95% CI, 4.60-4.72; Table 4).

**Table 4:** Additive likelihood ratios for having DISH considering BMI, DM, CE and OW

BMI	Diabetes	CE (cm)	OW (cm)	DISH probability	OR	95% CI
>30	Yes	≤2.5	≤2.5	0.5357	1.15	1.08-1.23
>30	Yes	≤2.5	2.5-5.0	0.7930	3.83	3.77-3.89
>30	Yes	≤2.5	>5	0.8232	4.66	4.60-4.72
>30	No	≤2.5	≤2.5	0.2884	0.41	0.34-0.48
>30	No	≤2.5	2.5-5.0	0.5736	1.35	1.27-1.42
>30	No	≤2.5	>5	0.4803	0.92	0.85-1.00
≤30	No	>2.5	≤2.5	0.4648	0.87	0.79-0.95
≤30	No	>2.5	2.5-5.0	0.7425	2.88	2.82-2.95
≤30	No	>2.5	>5	0.6644	1.98	1.91-2.05

BMI, body mass index; CE, chest expansion; DISH, diffuse idiopathic skeletal hyperostosis; DM, diabetes mellitus; OR, odds ratio; OW, occiput-to-wall distance.

## DISCUSSION

We present an analysis of clinical parameters from patients with DISH, collected in a clinical setting, in comparison to patients with back pain without DISH. One main result is that comparing CE of DISH with non-DISH patients, a CE of ≤2.5 cm was associated more with DISH, with high accuracy, sensitivity and specificity. This was true for both the whole non-DISH control group and for 41 patients of those with symptomatic back pain. In addition, OW distance was also significantly increased in patients with DISH compared with non-DISH (5.17 ± 3.26 cm vs 2.69 ± 3.3 cm, *P* < .001).

The reduced CE found in our study reflects and highlights the involvement of the thoracic cage as the preferable site of involvement in DISH [8,10]. Hyperostosis of the thoracolumbar articulation might be the explanation for the reduced CE, as well as well-known anterior chest ossifications [11,12]. A recent series even showed a very high frequency of DISH among hypoventilation syndrome, which is defined by a reduction in chest volumes [13]. As a limitation of CE is also well-described in patients with axial spondyloarthritis (axSpA) [14], its examination is part of the clinical routine in axSpA. Differentiating between DISH and axSpA can be challenging, especially considering that back pain is predominantly encountered in primary care rather than in expert rheumatology units, where female patients are more common. Therefore, a careful workup is essential for accurate diagnosis [15]. It should be noted, however, that CE, despite being a simple measurement, is not commonly used in routine physical examinations of patients with degenerative spinal diseases, including DISH and spondylosis. We suggest incorporating CE measurements in the evaluation of patients with DISH, as a reduction in CE may have clinical implications for these patients.

Smoking and the resulting decline in lung function may contribute to the reduction of CE. A study by Oudkerk demonstrated reduced lung volumes in patients with DISH compared with those without DISH in a large cohort of lung cancer screening CT scans, nearly half of whom were smokers [16]. Therefore, the potential reduction in CE among smokers with DISH should be considered during their clinical assessment. The association of DISH with metabolic syndrome and/or other constitutional measures has been previously described [[17], [18], [19]]. We found that the OR for having DISH among patients with (all of those conditions together): BMI ≥30, type 2 DM, CE ≤2.5 cm and OW ≥5 cm was 4.66. These results confirm the long-observed associations between ectopic thoracic spine bone formation and systemic inflammatory conditions such as metabolic syndrome or obesity. As already known, severe obesity also mechanistically results in reduced lung volumes due to increased load on the muscles of the chest cage. Here, it might be that the increased bone formation associated with metabolic syndrome contributes more to the pathophysiological mechanism of reduced CE. The results of restrictive lung function may be associated with an elevated risk of cardiovascular outcomes, as seen in hypoventilation syndrome and obstructive sleep apnoea both described in DISH population [7,13,20]. As DISH is associated with obesity, it is possible that the presence of DISH only reflects longstanding obesity and does not have a direct effect on CE. Hence, the cause for lower CE seen in patients with DISH might also be the direct effect of obesity (ie, higher BMI) on respiratory mechanics. The probability of DISH in obese and diabetic individuals is high, yet CE was ≤2.5 cm and high OW distance, add for the likelihood of DISH, independently.

We therefore suggest that the addition of CE and OW measurements might prove useful in earlier recognition of DISH in the elderly population and in those with metabolic syndrome.

A limitation of our study is a possible bias arising from the different sex distribution and the higher proportion of female patients found in the DISH group. We believe that the reason for this is the relatively high referral of females to the rheumatic disease unit, mainly for musculoskeletal complaints. DISH is found and diagnosed through a routine clinical workout and chest imaging, However, since there was no intent selection of patients to be included and all data were taken from clinical records, we matched the non-DISH in the same proportion of sex, thus mitigating the effect of the abnormal sex distribution.

## Conclusion

In comparison to non-DISH, the combination of high BMI, DM, low CE and high OW distance is highly suggestive of DISH compared with non-DISH with symptomatic back pain. CE and OW are simple physical examination measures that can be easily employed in a routine clinical setting. Future studies of these measures are warranted in larger cohorts of patients and with DISH patients in various levels of evolvement.

## Competing interests

The authors declare no conflicts of interest.
